# *MicroRNA-146a* Protects against Hepatocellular Carcinoma through Suppression of CCL5

**DOI:** 10.1158/2767-9764.CRC-25-0474

**Published:** 2026-02-20

**Authors:** Morgan C. Nelson, Liam C. O’Malley, Soh-Hyun Lee, Kaylyn M. Bauer, Arevik Ghazaryan, William W. Tang, Chad VanSant-Webb, Van B. Tran, Colton Hernandez, Ben Battistone, Amber Thibeaux, June L. Round, Micah J. Drummond, H. Atakan Ekiz, Kimberley J. Evason, Warren P. Voth, Ryan M. O’Connell

**Affiliations:** 1 Division of Microbiology and Immunology, Department of Pathology, University of Utah, Salt Lake City, Utah.; 2 Hunstman Cancer Institute, University of Utah, Salt Lake City, Utah.; 3 Department of Physical Therapy and Athletic Training, University of Utah, Salt Lake City, Utah.; 4 Department of Molecular Biology and Genetics, Izmir Institute of Technology, Urla, Turkey.

## Abstract

The miRNA *microRNA-146a* (*miR-146a*) regulates several aspects of chronic inflammation, including the hepatocellular carcinoma (HCC) risk factor, steatohepatitis. In this study, we find that the loss of *miR-146a* leads to a significantly increased tumor burden over a 9-month time frame in a mouse model of HCC. Notably, this *miR-146a*^*−/−*^ phenotype is most pronounced in females, who are typically not sensitive to this model. Mechanistically, we identified increases in dysfunctional CD8^+^ T cells that express high levels of CCL5 and resemble age-associated T (Taa) cells, as well as elevated levels of myeloid cells with a myeloid-derived suppressor cell (MDSC) phenotype, a class of myeloid cells that suppresses tumor immunity. Deletion of *Ccl5* from *miR-146a*–deficient mice returned tumor growth and the aberrant myeloid cell populations to wild-type levels. Surprisingly, deletion of *Ccl5* did not rescue the gross metabolic phenotype observed in *miR-146a*^*−/−*^ mice subjected to HCC induction, indicating that miR-146a plays an independent role in regulating HCC and metabolic disease. Taken together, this work reveals a critical host-protective role for *miR-146a* in HCC that is mechanistically dependent on CCL5 and wherein tumor burden correlates with two suppressive immune populations, providing an impetus for targeting these pathways to combat HCC. Furthermore, the correlations with tumor burden and Taa cells suggest that aging may increase HCC risk through the accumulation of CCL5-expressing Taa cells.

**Significance:**

The findings in this study highlight the potential of a miRNA-based therapy in the treatment of, and as a biomarker for, liver cancer, something especially promising given the liver’s avid uptake of RNA. Additionally, this work reveals possible causal roles in liver cancer for two unique immune cell types that are prevented by miR-146a.

## Introduction

Liver cancers account for more than 700,000 deaths worldwide each year (1), and hepatocellular carcinoma (HCC) is the most common form of primary liver cancer (2, 3). Several liver conditions are thought to contribute to HCC risk, such as metabolic dysfunction-associated steatotic liver disease (MASLD), which is estimated to affect more than 30% of adults (4). MASLD is the initial stage of an inflammatory process that may progress to metabolic dysfunction-associated steatohepatitis (MASH) and ultimately lead to cirrhosis (5). About 80% of HCC cases present with cirrhosis (6), but the etiology of this disease progression is still unclear. Growing evidence suggests that inflammation plays a crucial role in the development of MASH, cirrhosis, and, ultimately, HCC (7). Additionally, adverse immune responses play a crucial role in inflammation-driven hepatic tissue damage, which is a significant risk factor for HCC.

miRNAs are small noncoding RNA molecules that specifically inhibit translation or lead to the degradation of their target mRNAs (8). Previous work has shown that *microRNA-146a* (*miR-146a*), in particular, protects mice from diet-induced obesity and chronic steatohepatitis (9). This anti-inflammatory miRNA is activated by the NF-κB signaling pathway and functions to repress several known targets, thereby resolving inflammation (10). Additionally, dysregulated *miR-146a* expression is associated with many inflammation-driven diseases (11–13). The clinical presentation of HCC has been correlated with both functional polymorphisms and a decrease in *miR-146a* in cancerous liver tissue in our work (Supplementary Fig. S1) and others (14–16). Furthermore, NF-κB–driven inflammation has been shown to directly promote HCC (17).

Because the liver is constantly exposed to potentially pathogenic immune activators, it exhibits a tolerogenic immune environment during homeostasis (18). This status includes incomplete T-cell activation, tolerogenic myeloid cell populations, immunosuppressive cytokine secretion, and immune checkpoint expression (19). During progression to HCC, certain protumor immune populations expand further, including regulatory CD4^+^ T cells, exhausted CD8^+^ T cells, myeloid-derived suppressor cells (MDSC), as well as tumor-associated and M2-like macrophages (20).

CCL5 is a chemokine that can promote tumor growth by recruiting immune cells to sites of injury and inducing immunosuppressive phenotypes upon recruitment. *Ccl5* expression is initiated as part of the NF-κB and JAK/Stat signaling pathways (21), and *miR-146a* has been shown to directly target *Ccl5* in human macrophages (22, 23). Heightened CCL5 has been identified in many cancers and can be secreted by both tumor and nontumor cells, including CD8^+^ T cells (24).

Here, we provide evidence that *miR-146a* constrains HCC tumor growth in mice by regulating CCL5 expression. HCC was induced in these mice by injection of the chemical carcinogen N-diethylnitrosamine (DEN, N-nitrosodiethylamine) at 14 days of age. A week later, the mice were weaned and placed on a choline-deficient high-fat diet (CDHFD) to induce fibrosis and hasten the onset of disease. In the absence of *miR-146a*, an elevated, dysfunctional CD8^+^ T-cell population resembling age-associated CD8^+^ (Taa) cells, which express high levels of CCL5, was present. Furthermore, increased recruitment of myeloid cells with an MDSC phenotype was observed in *miR-146a*^*−/−*^ DEN-CDHFD–treated mice. Deletion of *Ccl5* in the context of a *miR-146a* deficiency reverses tumor growth and immunosuppressive cell phenotypes, confirming that CCL5 is essential for elevated tumor burden and further suggesting that these immune populations of interest may play a role but do not affect the gross metabolic phenotype observed in this model.

## Materials and Methods

### Sex as a biological variable

Initial mouse experiments were conducted in male and female mice. Given the differing results, certain experiments were only conducted in females, in which larger differences in phenotype were observed. The sex of each animal in each experiment is noted in the figure legends, and where appropriate, additional data for both male and female mice are provided in the supplemental figures.

### Animal maintenance and treatment

All wild-type (WT; RRID: IMSR_JAX:000664), *miR-146a*^*−/−*^ (RRID: IMSR_JAX:016239), and *Ccl5*^*−/−*^ (RRID: IMSR_JAX:005090) mice were on a C57BL/6 background in a specific pathogen-free mouse facility at the University of Utah, USA, in accordance with approved Institutional Animal Care and Use Committee (IACUC) protocols. Experimental HCC mice were given i.p. injections at 14 days old of 25 mg/kg DEN (Sigma-Aldrich, #N0756-10ML). HCC mice were weaned at 21 days and placed on a 60% CDHFD (Research Diets, # D05010403i). HCC mice were monitored for the duration of the study; mice were weighed at specified times, and cheek bleeds were taken to collect blood at indicated times. HCC mice were sacrificed at 32 to 36 weeks for endpoint analysis.

Aging, cancer-free mice (*miR-146a*^*−/−*^ and WT) were maintained on a standard diet and sacrificed at 43 to 60 weeks for analysis.

### Alanine aminotransferase and alpha-fetoprotein assays

Blood was collected by cheek bleed at approximately 8, 19, 26, and 34 weeks of age. To collect serum, the blood was incubated at room temperature for 2 hours and then spun at 2,000 × *g* for 20 minutes. For alpha-fetoprotein (AFP) ELISAs (R&D Systems, #MAFP00), serum was diluted 20- to 100-fold in the diluent provided in the kit, and the assay directions were followed. ELISA plates were read at 450 nm + 570 nm on a plate reader. For alanine aminotransferase (ALT) assays (Sigma-Aldrich, #MAK052-1KT), 5 to 10 μL of serum was used, and the assay directions were followed.

### HCC assessment

Mice were sacrificed in a CO_2_ chamber, and cervical dislocation was used as a second method of euthanasia. Serum was collected directly following sacrifice, and spleens and livers were taken for further analysis. Livers were perfused with 30 mL of ice-cold PBS before removal from the mice. Subsequently, they were weighed and photographed, and the visible tumor burden was then counted on each liver. Right lobes were taken for histologic analysis and fixed in 4% formalin overnight and then embedded in paraffin and stained with Masson’s trichrome or hematoxylin and eosin (H&E). Sections of healthy liver or tumor were flash-frozen for RNA and protein analysis. For RNA, tissue was processed in QIAzol (QIAGEN, cat. #79306), and total RNA was collected using the miRNeasy mini kit (QIAGEN, cat. #217004) for qRT-PCR analysis. qPCR primer sequences and/or catalog numbers are listed in Supplementary Table S1. The remaining livers were processed for analysis by flow cytometry.

### Hepatocyte Isolation

Hepatocytes were isolated from mouse livers perfused with 30 mL of ice-cold PBS. The liver was then rinsed in PBS and cut up with a razor. This PBS-liver mix was filtered through a 70 μm filter into 20 mL of Percoll–Hank’s Balanced Salt Solution [HBSS; 2 mL of 1× HBSS and 18 mL of 1× Percoll (Cytiva, cat. #17089109)] in a 50 mL conical tube. The tube was inverted 20 times to mix and then centrifuged at 300 *g* for 10 minutes at 4°C. The supernatant was aspirated off, and 30 mL of ice-cold PBS was added to the pellet. This was followed by a second centrifugation at 300 *g* for 10 minutes at 4°C. The supernatant was again aspirated, and the pellet was resuspended in 25 mL PBS with 10% FBS and 1% penicillin-streptomycin, followed by a final spin of 300 *g* for 10 minutes at 4°C. The supernatant was again aspirated, and the pellet was resuspended in 700 μL QIAzol and frozen for future RNA extraction and qPCR analysis.

### Histologic analysis

The right lobe of each liver was fixed within a histology cassette overnight at room temperature in 10% buffered formalin phosphate solution and then transferred to 70% EtOH at 4°C until staining. Lobes were longitudinally sectioned; then H&E staining and trichrome staining were completed. All histologic processing/staining was performed at the Huntsman Cancer Institute Biorepository and Molecular Pathology Resource core in the ARUP-operated Research Histology division. Hepatic scoring and microscopic tumor counting were conducted by a blinded pathologist, as described in the figure legends.

### Flow cytometry and FACS isolation

Hepatic leukocytes were isolated from sections of perfused livers using the following procedure. Minced tissue was digested for 25 minutes in RPMI with 0.5 mg/mL collagenase, gently crushed through 100 μmol/L nylon mesh cell strainers, pelleted by centrifugation at 300 × *g* for 5 minutes, washed in HBSS and then PBS, and centrifuged at 1,000 × *g* for 30 minutes from a 30% Percoll (Cytiva, cat. #17089109) solution underlaid with a 70% Percoll solution. The layer of cells at the interface (leukocytes) was recovered and washed in PBS. Samples were first Fc receptor blocked with CD16/32 antibodies (BioLegend, cat. #101330, RRID:AB_2561482) for 20 minutes at 4°C. Cells were pelleted after this and every step by centrifugation at 400 × *g* for 5 minutes. Splenocytes were prepared identically, except there was no tissue digestion.

Cells were stained with the BioLegend Zombie UV Fixable Viability Kit (BioLegend, #423107) for 20 minutes at room temperature. Cells were then stained with surface marker antibodies for 30 minutes in the dark at 4°C. The antibodies used varied between experiments. The antibodies used, along with their corresponding RRIDs, are listed in Supplementary Table S2. After staining, cells were washed 2 times for 5 minutes each at room temperature with column buffer (1× HBSS without Ca or Mg, containing 10 mmol/L HEPES, 5 mmol/L EDTA, and FBS). Cells were fixed overnight with 1% paraformaldehyde if no intracellular staining was needed and then washed in column buffer 2× before running on the flow cytometer. If intracellular staining was necessary, the FoxP3/Transcription Factor Staining Buffer kit (Tonbo Biosciences) and protocol were followed. For experiments in which CCL5 was stained (Supplementary Fig. S7), cells were incubated at 37°C with BD GolgiPlug Protein Transport Inhibitor (cat. #51-2301KZ; 1 μL GolgiPlug/1 mL of media) for 2 hours prior to staining with the Zombie dye. Cells were then stained with the Zombie dye and followed through the procedure described above. All samples were then resuspended in 300 μL of column buffer and run on a BD LSRFortessa (RRID: SCR_018655; Figs. 4, 5D, E, and 6E–G; Supplementary Fig. S5) or Cytek Aurora (RRID: SCR_019826; Fig. 5I and J; Supplementary Fig. S7) after compensating with UltraComp eBeads (Thermo Fisher Scientific, cat. #01-2222-42) and using single-stain controls. Samples for FACS purification of T-cell subtypes for RT-qPCR were prepared as above using CD45-PacBlue (BioLegend, cat. #103126, RRID:AB_493535), CD3-PE (BioLegend, cat. #100308, RRID:AB_312673), CD4-PerCP (BioLegend, cat. #100538, RRID:AB_893325), and CD8-APC (BioLegend, cat. #100712, RRID:AB_312751) antibodies, or CD45-FITC (BioLegend cat. #103108, RRID:AB_312973)for single-cell RNA sequencing (scRNA-seq) of all immune cells. FlowJo was used to analyze flow cytometry data.

### RT-qPCR analysis

Approximately 0.1 g of nontumor liver was collected from mice. RNA was extracted from these tissues using miRNeasy kits (QIAGEN, cat. #217004). When conventional mRNA targets were measured, cDNA was synthesized using the qScript cDNA Synthesis Kit (Quantabio, cat. #95048-025) and PowerUp SYBR Green Master Mix (Thermo Fisher Scientific, cat. #A25742). The LC480 (Roche LightCycler 480 Real-Time PCR System, RRID:SCR_018626) was used for qPCR to measure the expression of various genes. When miRNA targets were measured, the QIAGEN miRCURY LNA RT kit (cat. #339340) was used to make cDNA, and for qPCR, the QIAGEN miRCURY LNA SYBR Green PCR Kit (cat. #339347) and the Applied Biosystems QuantStudio 6 Flex instrument (RRID:SCR_020239) were used. Primer set sequences or catalog numbers and vendor details are listed in Supplementary Table S1.

### ELISAs

IL6 ELISAs were performed according to the manufacturer’s protocol (Thermo Fisher Scientific, cat. #88-7064-22).

### scRNA-seq

Sample preparation and analysis were conducted as described by Tang and colleagues (25). Mouse liver was processed into a single-cell suspension and then stained with DAPI and FITC-conjugated anti-CD45 antibody. Following staining, cells were washed twice with 1× DPBS containing 0.4% BSA (Miltenyi Biotec) and then FACS-sorted on the BD FACSAria Cell Sorter (RRID:SCR_016695). Sorted cells were washed in 1× DPBS with 0.4% BSA and processed for scRNA-seq by the High Throughput Genomics (HTG) Core. The scRNA-seq was performed on the 10x platform according to the manufacturer’s instructions and sequenced on the NovaSeq 6000 (RRID:SCR_016387). Gene reads were processed with the 10x Genomics Cell Reports 44, 115301, February 25, 2025, 23 Article II OPEN ACCESS Ranger pipeline [tenx (RRID:SCR_016957)]. Before analysis, mitochondrial gene representation and the variance of unique molecular identifier counts were regressed out. The FastQ files were aligned with the refdata-gex-mm10-2020-A mouse reference dataset from 10× Genomics using CellRanger Count version 5.0.0, and feature-barcode matrices were created. Following this step, analyses were conducted using the Seurat R package (version 4.2.0, RRID:SCR_016341). Reprocessing of data to remove low-quality cells was performed based on the following criteria: Cells with fewer than 600 features (considered low-quality), more than 6000 features (potentially indicating duplicates), or a mitochondrial gene fraction greater than 5% (indicative of stressed or dying cells) were excluded from further analysis. These values were determined by examining the general distribution in the data, and they are consistent with prior work done by us and others (25). After removing these cells from the dataset, Seurat’s sctransform method normalized and integrated the datasets. Next, dimensionality reduction was performed through principal component analysis and Uniform Manifold Approximation and Projection for Dimension Reduction (UMAP) with 11 principal components. Single cells were clustered based on nearest-neighbor graph construction using the FindNeighbors and FindClusters Seurat functions, and 16 clusters were identified at the resolution level of 0.2. Marker genes specific to each cluster were identified using Seurat’s FindAllMarkers function. To name single-cell clusters, genome-wide expression profiles were checked against the ImmGen reference samples using Spearman’s correlation method of the CIPR R package (RRID:SCR_027697), as defined previously (26). Cluster naming was also assessed by examining the expression profiles of known marker genes. Differences at the cluster level were visualized by determining the proportions of each cluster in each individual sample. Differential expression analyses between sample groups were performed at the cluster level using the FindMarkers() function of Seurat with default parameters and no log fold change threshold.

### miRNA expression analysis in the patient’s liver

To determine significantly dysregulated miRNAs in patients with MASH and MASH-derived HCC, we used the NanoString nCounter Human v3 miRNA Expression Assay kit (CSO-MIR3-12). In total, samples from four patients with MASH and no clinical evidence of HCC, and eight patients with HCC, were obtained from the University of Utah Pathology Archives. As controls, samples from four patients without underlying chronic liver disease, who had partial resections for benign liver tumors or metastases, were used. Patients were excluded if they had a history of prior infection with hepatitis B or C viruses or a documented clinical history of alcohol use. The University of Utah HTG Shared Resource extracted RNA from formalin-fixed, paraffin-embedded samples and processed it using the nCounter Human v3 miRNA Sample Prep and Expression Assay Kit. miRNA counts were analyzed using nSolver, and their expression levels were then validated via qPCR (Supplementary Fig. S1). This study was reviewed and approved by the University of Utah Institutional Review Board (IRB) and determined to be exempt from human subjects’ research (IRB_00091019). Therefore, informed consent is not applicable to this research on patient samples.

### Quantification and statistical analysis

GraphPad Prism software (RRID:SCR_002798) was used for graphing and statistical analysis of experimental data. Two-tailed Student *t* tests were used to calculate *P* values unless otherwise noted in figure legends. Quantitative data are displayed as mean ± SEM. *P* values are shown as indicated: *, ≤0.05; **, ≤0.01; ***, ≤0.001; ns, *P* > 0.05.

### Study approval

Mouse experiments were approved by the University of Utah’s IACUC. Patient studies were approved by the University of Utah’s IRB.

## Results

### *miR-146a* protects mice from DEN-induced HCC

**Figure 1.**
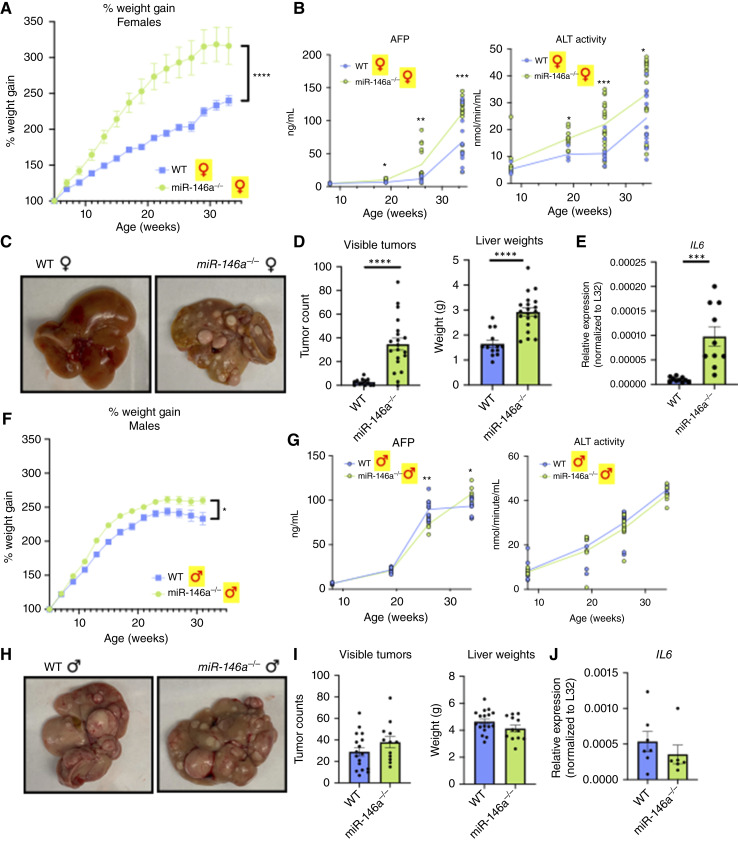
*miR-146a* protects females from DEN-induced HCC but is not fully sufficient to protect males. **A,** Loss of *miR-146a* reduces cumulative weight gain over 35 weeks on CDHFD in DEN-treated females. **B,** Increased AFP and ALT markers of liver injury and HCC during the 35-week course on CDHFD in DEN-treated *miR-146a*^*−/−*^ females. **C,** Increased incidence of visible liver tumors and liver mass in *miR-146a*^*−/−*^ females after 35 weeks on CDHFD with DEN treatment, quantified in **D**. **E,** Increased IL6 expression by qRT-PCR in livers from female mice in the absence of *miR-146a*. **F,** Percentage of weight gain over 35 weeks on CDHFD in DEN-treated WT or *miR-146a*^*−/−*^ males. **G,** AFP and ALT markers of liver injury and HCC during the 35-week course on CDHFD in DEN-treated WT or *miR-146a*^*−/−*^ males. **H,** Incidence of visible liver tumors and liver mass in WT or *miR-146a*^*−/−*^ males after 35 weeks on CDHFD with DEN treatment, quantified in **I**. **J,** Liver IL6 expression in WT or *miR-146a*^*−/−*^ males. Data represent three experiments; percentage of weight gain line graphs display mean ± SEM (**A** and **F**) for 22 *miR-146a*^*−/−*^ mice and 13 WT mice or 12 *miR-146a*^*−/−*^ and 19 WT mice, respectively. AFP and ALT line graphs display points representing each mouse, with means connected by lines (**B** and **G**). Bar charts display points representing each mouse and error bars that are mean ± SEM. In all charts, *, *P* < 0.05; **, *P* < 0.01; ***, *P* < 0.001; ****, *P* < 0.0001.

We previously reported that *miR-146a*^*−/−*^ mice exhibit hepatosteatosis, a known predisposing factor for HCC, when placed on a high-fat diet (9). Additionally, we found that *miR-146a* levels were lower in cancerous liver tissue of patients compared with adjacent noncancerous liver tissue and that they were also lower in the livers of patients with MASH-cirrhosis (no HCC) compared with healthy patients (Supplementary Fig. S1). We thus tested HCC development in *miR-146a*^*−/−*^ mice compared with WT controls. Mice were treated with DEN-CDHFD or with a PBS vehicle control and CDHFD. Over 35 weeks, *miR-146a*^*−/−*^ mice gained significantly more weight than WT controls, regardless of DEN or PBS administration (Fig. 1A and F; Supplementary Fig. S2A and S2F). During this time course, serum levels of AFP, in which expression coincides with liver cancer, increased in female *miR-146a*^*−/−*^ mice compared with WT. Serum ALT activity, which marks hepatocellular injury, also increased in *miR-146a*^*−/−*^ females (Fig. 1B; Supplementary Fig. S2B). Male *miR-146a*^*−/−*^ mice exhibited no overall differences versus WT in AFP levels or ALT activity in DEN-injected mice (Fig. 1G; Supplementary Fig. S2G).

Upon harvest, tumor burden was significantly higher in *miR-146a*^*−/−*^ female mice than in WT female mice, and *miR-146a*^*−/−*^ females had larger livers (Fig. 1C and D). No such differences were observed between *miR-146a*^*−/−*^ and WT males, as all males were heavily tumor-laden at this time point (Fig. 1H and I). However, during subsequent experiments, we found that male *miR-146a*^*−/−*^ mice had a significantly increased tumor burden at 7 months of age, an earlier time point, suggesting that *miR-146a*’s protective effect is still at play in males (Supplementary Fig. S3). No significant tumor differences were observed in male or female vehicle-treated mice, with few, if any, tumors developing (Supplementary Fig. S2C, S2D, S2H, and S2I).

Previous work has shown that males have greater DEN-induced serum IL6 production than females, which corresponds with the higher frequency of HCC in males. Moreover, this sex difference in HCC can be reversed with IL6 ablation (27). Thus, the sex-specific differences seen in our study may be due, at least in part, to differential IL6 production. IL6 is regulated by *miR-146a* through NF-κB signaling, so we expected IL6 derepression in *miR-146a*^*−/−*^ mice compared with WT. Although males had overall higher *IL6* levels than females, the differences between genotypes were insignificant in males (Fig. 1J). In contrast, female *IL6* levels were significantly higher in *miR-146a*^*−/−*^ mice than in WT (Fig. 1E). This correlates with the observed differences in tumor burden, in which males have an overall greater burden than females, but only females showed significant genotype-specific differences. The remainder of this work thus primarily focuses on HCC in females, with the novel observations from our system contributing new insights into female-specific differences in HCC.

### Inflammation and tumor burden are exacerbated in *miR-146a*^*−/−*^ mice

**Figure 2.**
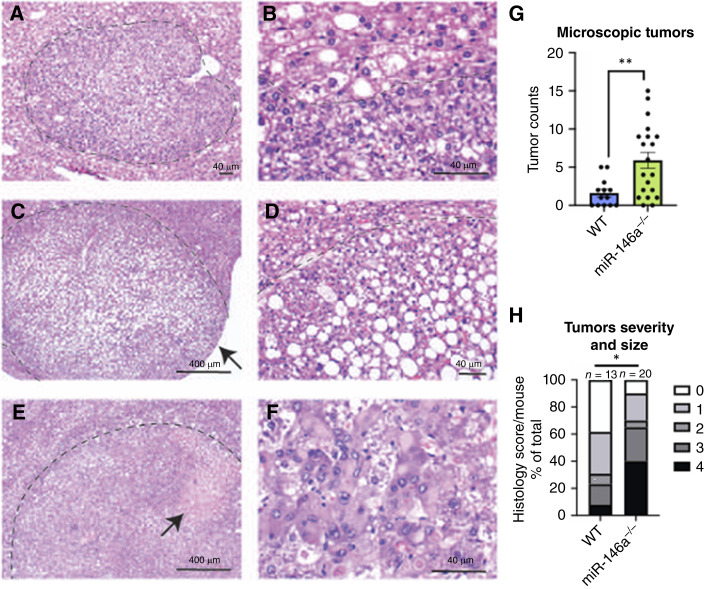
Examples of lesions in DEN-treated WT and *miR-146a*^*−/−*^ female mouse livers. **A** and **B,** Focus of cytologic alteration (FCA). At 10× (**A**), the lesion appears as a small (less than 2 mm) zone of more darkly staining hepatocytes. At 40×, nonlesional hepatocytes at the periphery of the lesion (top) appear larger than cells within the FCA (bottom). **C** and **D,** Hepatocellular adenoma (HCA). At 4× (**C**), the tumor can be seen to distort the liver capsule (arrow), and no normal portal tracts are seen within the lesion. At 20× (**D**), nonlesional hepatocytes at the periphery of the lesion (top left) are compressed. **E** and **F,** HCC. At 4× (**E**), the tumor shows a zone of necrosis (arrow). The lesion lacks normal portal tracts, and the surrounding nonlesional hepatocytes are compressed. Nuclear enlargement, nuclear contour irregularities, increased nuclear-to-cytoplasmic ratios, and architectural disarray are seen at 40× (**F**). H&E stains. Scale bars are 40 μm (**A**, **B**, **D**, and **F**) or 400 μm (**C** and **E**). **G,** Microscopic tumors were counted from one right lobe of each liver. **H,** Tumor severity and size. 0 = no tumor; 1 = FCA ≤1 mm; 2 = FCA >1 mm; 3 = HCC or HCA ≤2 mm; 4 = HCC or HCA >2 mm (*Fisher’s exact test separated between HCA/HCC and non-HCA/HCC). Data represent three experiments. The bar chart (**G**) displays points representing individual mice and error bars representing mean ± SEM. *, *P* < 0.05; **, *P* < 0.01.

Histologic examination of livers from WT and *miR-146a*^*−/−*^ mice at the experimental endpoint revealed many lesion features, including foci of cytologic alteration (Fig. 2A and B), hepatocellular adenoma (Fig. 2C and D), and HCC (Fig. 2E and F). When scored histologically, DEN-CDHFD–treated *miR-146a*^*−/−*^ mice had more microscopic tumors per mouse than WTs (Fig. 2G), and the tumors were larger and in a more advanced stage (Fig. 2H). Males showed no difference in tumor count, size, or severity at this same 35-week time point (Supplementary Fig. S4A and S4B). Mice that received vehicle injections exhibited no tumors (Supplementary Fig. S4G and S4H).

**Figure 3.**
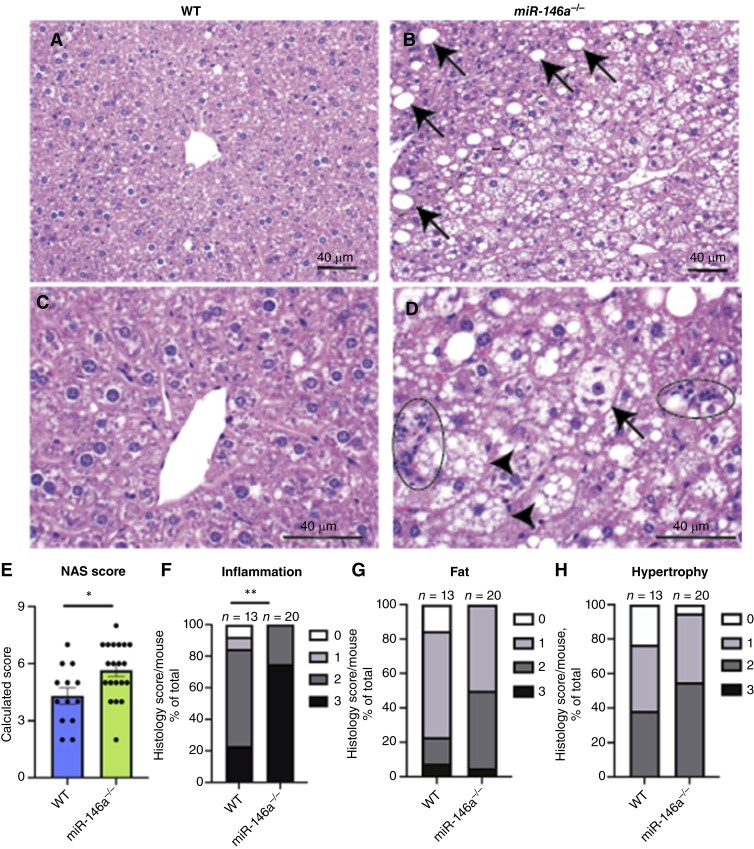
Steatohepatitis in nontumor regions of DEN-treated WT and *miR-146a*^*−/−*^ female mouse livers. **A,** WT mouse liver, showing no significant steatosis, hypertrophy, or inflammation. **B,***miR-146a*^*−/−*^ liver, showing macrovesicular steatosis (arrows) and zones of microvesicular steatosis and hypertrophy (lower right of dashed line). **C,** WT mouse liver, showing a central vein with surrounding unremarkable hepatocytes. **D,***miR-146a*^*−/−*^ genotype, showing two clusters of inflammatory cells (circled) and hypertrophy. Some hypertrophied hepatocytes contain microvesicular steatosis (arrowheads), whereas others have rarified cytoplasm and Mallory–Denk bodies similar to ballooned hepatocytes in human patients (arrow). H&E stains. Scale bars are 40 μm. **A** and **B,** Magnification 20×. **C** and **D,** Magnification 40×. **E,** Non-alcoholic fatty liver disease activity scores (NAS). Scores were calculated on a nine-point scale with the nine points being derived from a sum of these measurements: hepatocyte hypertrophy, inflammation, and steatosis using an established scoring system (52). **F,** Inflammation score (Fisher’s exact test, separated between 0–2 and 3). **G,** Fat cores: 0 ≤ 5% fat; 1 = 5%–33% fat; 2 = 33%–66% fat; 3 ≥ 66% fat. **H,** Hypertrophy score (Fisher’s exact test, separated between 0–2 and 3). The data represent three experiments. Bar chart (**E**) displays points representing individual mice and error bars representing mean ± SEM. *, *P* < 0.05; **, *P* < 0.01.

Histology also revealed nontumor hepatic features of MASH compared with a normal healthy liver (Fig. 3A and C), including steatosis and hypertrophy (Fig. 3B) and inflammatory cell infiltration (Fig. 3D). *miR-146a*^*−/−*^ female animals exhibited more advanced-stage non-alcoholic fatty liver disease activity score (NAS) (Fig. 3E; Supplementary Fig. S3G), which was determined by the summation of histologic inflammation, hypertrophy, and fat scores (Fig. 3F–H; Supplementary Fig. S4G). Males exhibited no significant genotype differences at this time point (Supplementary Fig. S4C–S4F and S4H). Female *miR-146a*^*−/−*^ exhibited more severe disease progression than WTs, indicating that *miR-146a* protects against lipid accumulation, steatosis, MASH, and tumor formation in females.

### Immune populations are altered in *miR-146a*^*−/−*^ livers during disease

**Figure 4.**
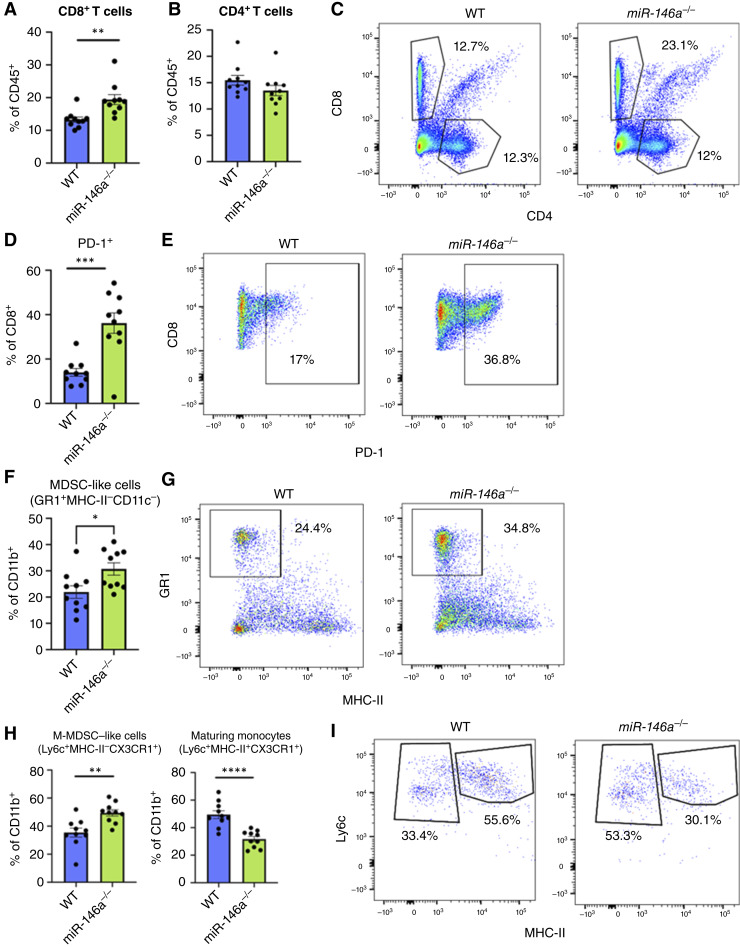
Tumor-bearing female *miR-146a*^*−/−*^ mouse livers contain increased PD-1^+^ CD8 T cells and increased aberrant myeloid populations. **A,** Significantly increased CD8^+^ T cells were seen in *miR-146a*^*−/−*^ livers although no significant change (**B**) in CD4^+^ T cells from *miR-146a*^*−/−*^ livers was observed. **C,** Representative flow plots for (**A**) and (**B**), showing CD4^+^ and CD8^+^ populations in CD45^+^ lymphocytes. **D** and **E,** Frequency and representative flow plots for PD-1^+^ CD8 T cells from WT and *miR-146a*^*−/−*^ liver lymphocytes. **F** and **G,** Frequency and representative flow plots for cells with the surface marker profile of MDSCs (MDSC-like) in the CD11b^+^ population from WT and *miR-146a*^*−/−*^ liver. **H** and **I,** Frequency of and representative flow plots of two populations of myeloid cells (M-MDSC–like cells and maturing monocytes). Bar charts contain dots representing individual mice, and error bars represent mean ± SEM. *, *P* < 0.05; **, *P* < 0.01; ***, *P* < 0.001; ****, *P* < 0.0001.

Inflammatory immune clusters were a prominent histologic feature in the livers of *miR-146a*^*−/−*^ mice (Fig. 3D), prompting further assessment of the immune populations present in these livers. To determine the presence of specific immune populations, we isolated immune cells from livers at the experimental endpoint. We observed elevated CD8^+^ T-cell percentages in *miR-146a*^*−/−*^ mice compared with WTs, whereas CD4^+^ T cells remained unchanged (Fig. 4A–C). A high percentage of CD8^+^ T cells in the *miR-146a*^*−/−*^ population were positive for PD-1, an inhibitory receptor often associated with an accumulation of exhausted effector CD8^+^ T cells (Fig. 4D and E). PD-1^+^ CD8 T cells correlate with poor clinical outcomes in HCC (28).

When looking at additional immune populations via flow cytometry, there were no differences in Kupffer cells or dendritic cells (Supplementary Fig. S5), but there was a buildup of both CD11b^+^GR1^+^MHC-II^−^CD11c^−^ cells and CD11b^+^CX3CR1^int^Ly6c^+^MHC-II^−^ cells, surface marker profiles associated with MDSCs and monocytic MDSCs (M-MDSC), respectively (17, 29). Additionally, a corresponding decrease in CD11b^+^CX3CR1^int^Ly6c^+^MHC-II^+^ maturing monocytes was found in the livers of *miR-146a*^*−/−*^ mice (Fig. 4F–I). MDSCs are a heterogeneous population that suppresses T-cell activation and fails to present antigens, comprising two subtypes: M-MDSCs and PMN-MDSCs. In our model, we have demonstrated that M-MDSCs are present at higher levels in *miR-146a*^*−/−*^ livers. MDSCs systematically expand in cancer and exert an immunosuppressive effect (17, 30). Together, our flow cytometry analysis indicates that immunosuppressive and exhausted-like immune cell types expand in *miR-146a*^*−/−*^ DEN-CDHFD–treated mice.

### CD8^+^ T cells highly express *Ccl5* in *miR-146a*^*−/−*^ mice

**Figure 5.**
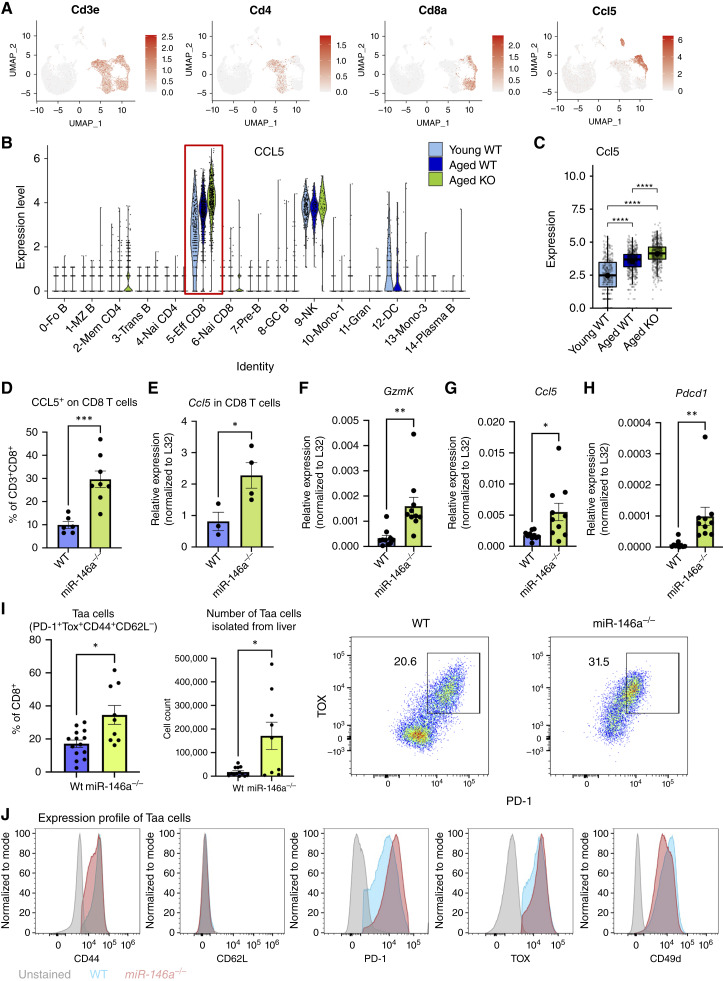
*Ccl5* is highly associated with aging *miR-146a*^*−/−*^ CD8^+^ T cells, and its expression is elevated in *miR-146a*^*−/−*^ male and female mice. **A,** scRNA-seq analysis of samples from young and aged (15 months) WT or *miR-146a*^*−/−*^ splenocytes from male and female mice using CIPR to annotate cell populations and to localize *Ccl5* expression in UMAP plot clusters. **B** and **C,** Increased *Ccl5* expression in aged *miR-146a*^*−/−*^ CD8^+^ effector T cells from the same mice as **A**. **D,** Flow analysis in CD8^+^ T cells shows increased CCL5 staining in female *miR-146a*^*−/−*^ liver. **E,** qRT-PCR for *Ccl5* expression in sorted CD8^+^ T cells from female WT or *miR-146a*^*−/−*^ splenocytes. **F–H,** qRT-PCR analysis of *Ccl5*, *Gzmk*, and *Pdcdc1* (PD-1), respectively, in livers from WT or *miR-146a*^*−/−*^ DEN-CDHFD–treated female mice. **I,** Quantification and representative flow plots of age-associated CD8 T cells (Taa cells) in the liver of male and female WT and *miR-146a*^*−/−*^ aged, cancer-free mice. **J,** Surface marker expression profile of Taa cells in male and female WT and *miR-146a*^*−/−*^ aged, cancer-free mice relative to an unstained liver sample. Bar charts contain dots representing individual mice, and error bars represent mean ± SEM. *, *P* < 0.05; **, *P* < 0.01; ***, *P* < 0.001; ****, *P* < 0.0001. scRNA-seq data have been uploaded to the NCBI Gene Expression Omnibus database (GSE138222).

Age-associated inflammation can significantly affect disease outcomes, and *miR-146a* is a critical regulator of inflammation and inflammatory disease (31, 32). To better understand the roles of immune populations that may be at play in our HCC studies, we analyzed scRNA-seq data from Ekiz and colleagues (33) using the CIPR analytic package on samples from young and aged WT or *miR-146a*^*−/−*^ splenocytes. UMAP cell type analysis reveals that *Ccl5* is predominantly expressed in cells most strongly associated with the *CD8a* expression pattern, identifying CD8^+^ T cells as potent expressors of *Ccl5* (Fig. 5A). NK cells were also high expressors of *Ccl5 in this dataset*, which may be of interest in follow-up studies, yet expression of miR-146a did not seem to affect *Ccl5* expression by NK cells. scRNA-seq data, which assign these splenocytes to 14 immune cell types, show that *Ccl5* is strongly expressed in all three groups of effector CD8^+^ T cells (Fig. 5B). Furthermore, *Ccl5* expression was significantly higher in aged *miR-146a*^*−/−*^ CD8^+^ T cells than in young or aged WTs by scRNA-seq (Fig. 5C), and this was corroborated by flow cytometry (Fig. 5D) and transcriptional analysis in CD8^+^ T cells (Fig. 5E) sorted from splenocytes.

One CD8 subtype known to express high levels of CCL5 is the Taa cell, which accumulates in aged tissue and promotes senescence within its environment. These T cells are part of the effector memory CD8 compartment and are characterized by heightened expression of granzyme K, PD-1, TOX, Eomes, and CD49d (34). In the splenic scRNA-seq effector CD8^+^ T-cell population, *Gzmk* (Granzyme K), *Pdcd1* (PD-1), *Tox*, and *Ccl5* were all elevated in *miR-146a*^*−/−*^ mice compared with WT (Supplementary Fig. S6A–S6D). To confirm the expansion of Taa cells, we performed a flow cytometry analysis on liver T-cell populations from cancer-free aged WT and *miR-146a*^*−/−*^ mice and identified the expansion of Taa cells in *miR-146a*^*−/−*^ livers, as indicated by higher levels of effector memory (CD44^+^ CD62L^−^) CD8^+^ T cells that are positive for PD-1, TOX, and CD49d (Fig. 5I and J).

Additionally, we examined the livers of DEN-CDHFD–treated mice at the endpoint and again found heightened *Ccl5* expression in *miR-146a*^*−/−*^ mice (Fig. 5G). These *Ccl5*-high livers also had heightened *Gzmk* (Fig. 5F) and *Pdcd1* (Fig. 5H), markers of Taa cells. Upon further examination of the DEN-CDHFD–treated livers from *miR-146a*^*−/−*^ mice, we confirmed not only that CD8 T cells express CCL5 but also that Taa cells display significantly higher expression of this protein compared with general CD8 T cells (Supplementary Fig. S7). Overall, this dataset shows that Taa cells are expanded in *miR-146a*^*−/−*^ mice and that CD8 T cells (particularly Taa cells) produce more CCL5 when *miR-146a* is absent. This positions CD8 T cells and Taa cells in particular as major producers of CCL5 in *miR-146a*-deficient mice, with CCL5 being a protein we will find to be essential for increased tumor burden.

### *Ccl5* is epistatic to *miR-146a* for tumor growth but not weight gain

The *Ccl5* transcript is directly downregulated by *miR-146a* in humans (22, 23), and it also responds to *miR-146a* regulation in mice. Secreted CCL5 can recruit MDSCs; further, its expression is associated with cancer progression (35, 36). Thus, we next sought to identify and characterize a *miR-146a*-CCL5 immune regulatory axis in DEN-CDHFD-instigated HCC.

**Figure 6.**
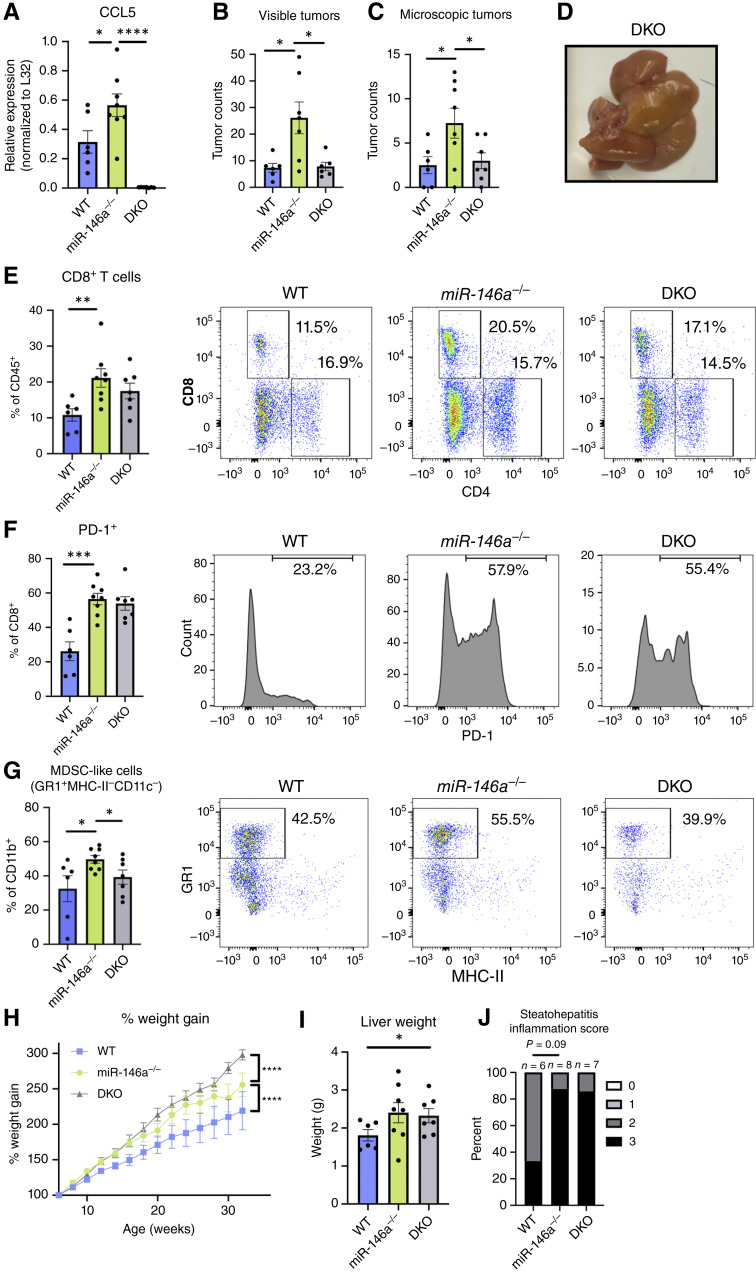
*miR-146a* ^*−/−*^ *Ccl5* ^*−/−*^ DKO female mice have lower tumor burden and MDSC-like cell infiltration, but greater weight gain than *miR-146a*^*−/−*^ female mice in DEN-CDHFD–instigated HCC. **A,** qRT-PCR for *Ccl5* expression in immune cells isolated via FACS with anti-CD45 antibody from WT or *miR-146a*^*−/−*^ mice and from *miR-146a*^*−/−*^*Ccl5*^*−/−*^ DKO mice to verify detection of *Ccl5*. **B–D,** Suppression of *miR-146a*^*−/−*^ macroscopic and microscopic tumor formation phenotype by deletion of *Ccl5* and overall liver morphology. **E** and **F,***miR-146a*^*−/−*^ CD8^+^ T-cell phenotypes after additional deletion of *Ccl5* and representative flow plots. **G,** Suppression of exacerbated MDSC-like cells from the loss of *miR-146a* by additional deletion of *Ccl5* and representative flow plot. **H,** Cumulative weight gain increases due to *miR-146a*^*−/−*^ in the DEN-CDHFD time course are not suppressed but are exacerbated by additional *Ccl5* mutation. **I** and **J,** Increased general non-HCC liver pathology in *miR-146a*^*−/−*^ does not require CCL5, as indicated by a lack of suppression of liver weight and steatohepatitis in DKO animals. Steatohepatitis inflammation scoring was conducted as described in Fig. 3F. Bar charts contain dots representing individual mice and error bars representing mean ± SEM. The percentage of weight gain line graph (**H**) displays points and error bars that are the mean ± SEM of 6 WT, 10 *miR-146a*^*−/−*^, and 7 DKO mice. *, *P* < 0.05; **, *P* < 0.01; ***, *P* < 0.001; ****, *P* < 0.0001. All data are from female mice only.

We generated double knockout mice deficient for both *miR-146a* and *Ccl5* (DKO) and challenged females with DEN, maintaining them on CDHFD, to determine whether CCL5 depletion could restore protection from HCC. At the endpoint of the experiment, we verified *Ccl5* deletion in liver immune cells purified via FACS sorting (Fig. 6A). We found that tumor burden was decreased in DKO mice compared with *miR-146a*^*−/−*^ alone, both visibly and histologically (Fig. 6B–D). The livers of DKO mice still contained CD8^+^ T cells with heightened PD-1 expression (Fig. 6F). However, these livers exhibited a lower percentage of the previously identified myeloid population displaying an MDSC phenotype (Fig. 6G), suggesting that recruitment of these leukocytes to the liver requires CCL5. This finding positions the elevated CD8^+^ population with high expression of CCL5, which resembles Taa cells, as a possible driver of disease and identifies a relevant pathway for potential therapeutic control of tumor growth in the clinic.

**Figure 7.**
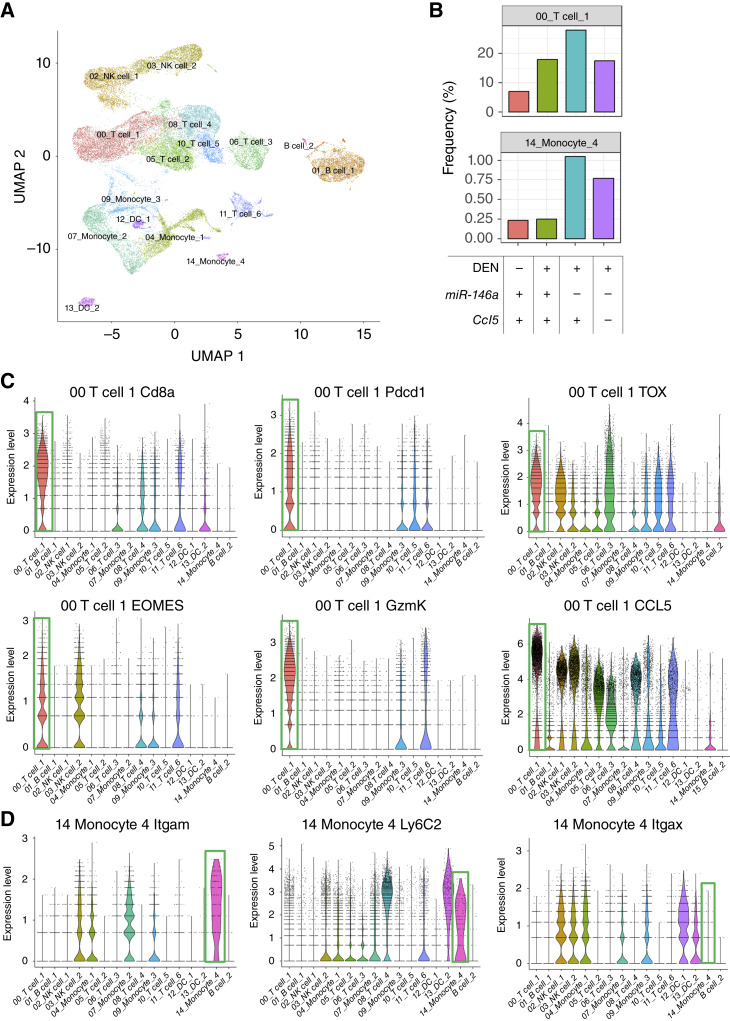
DEN-CDHFD–treated *miR-146a* female mice have an increased proportion of CCL5-expressing dysfunctional CD8 T cells and an aberrant monocyte population according to scRNA-seq. **A,** UMAP displaying annotated cell clusters revealed by scRNA-seq using mouse livers. **B,** The levels of a T cell and monocyte cluster in 2-month-old WT mice, DEN-CDHFD–treated WT mice, DEN-CDHFD–treated *miR-146a*^*−/−*^ mice, and DEN-CDHFD–treated *miR-146a*^*−/−*^; *Ccl5*^*−/−*^ mice (left to right). DEN-CDHFD–treated mice are 35 weeks old at the endpoint. **C,** T-cell 1 cluster is composed of CD8^+^ T cells expressing markers associated with Taa cells, including *Ccl5*. **D,** Myeloid markers identifying the monocyte 4 cluster, including *Itgam*, for which the monocyte 4 cluster has higher expression than other monocyte clusters. scRNA-seq data have been uploaded to the NCBI Gene Expression Omnibus database (GSE300674).

Additionally, we isolated CD45^+^ cells from the different groups to assess the impact of *miR-146a* and CCL5 on immune populations and their gene expression in DEN-CDHFD-treated tumor-bearing mice using 10x Genomics scRNA-seq. Data analysis with the CIPR package revealed an elevated T-cell and myeloid population in *miR-146a*^*−/−*^ mice and a reduced population in DKO mice (Fig. 7A and B). Further profiling of changes in other immune populations was also reviewed (Supplementary Fig. S8). The elevated T-cell population increased with the deletion of *miR-146a* and was identified as CD8^+^ T cells expressing high levels of PD-1. Thus, we believe this population is equivalent to the PD-1^+^ population originally identified via flow cytometry in DEN-CDHFD–treated female *miR-146a*^*−/−*^ mice (Fig. 4D). Additionally, this population exhibits high expression of *Ccl5* and several other genes that are markers of Taa cells (Fig. 7C), providing further evidence that the CCL5-secreting PD-1^+^ CD8 population elevated in *miR-146a*^*−/−*^ mice across experiments and methods are Taa cells. The myeloid population elevated in *miR-146a*^*−/−*^ mice and subsequently reduced in DKO mice were identified as monocytes, which expressed higher levels of the *Itgam gene* than the other three monocyte populations (Fig. 7D), suggesting that these cells may also be MDSCs (37–39).

Outside of differences in immune cell populations and tumor growth, DKO mice exhibit exacerbated weight gain compared with both WT and *miR-146a*^*−/−*^ mice (Fig. 6H), and their liver weights remain higher than those of the WTs (Fig. 6I). This finding uncouples the roles of *miR-146a* in protecting against fat accumulation and tumor progression. Although *miR-146a* deficiency leads to weight gain and inflammation in both the *miR-146a*^*−/−*^ mice and DKOs (Fig. 6H–J), *miR-146a* deficiency only leads to tumor progression when CCL5 is present. This suggests that *miR-146a*-CCL5–regulated immune involvement and recruitment may be an important factor in tumor progression, even in an obesogenic environment.

## Discussion

We found that *miR-146a* protects female mice from weight gain, tumor development and severity, MASH, and hepatic inflammation. This protection is clinically relevant given the decreases in *miR-146a* seen in liver tissue from patients with HCC (14, 16). *miR-146a* also protects against exacerbated CCL5 expression, including from dysfunctional CD8^+^ T cells that resemble Taa cells in the tumor microenvironment and are at increased levels in *miR-146a*^*−/−*^ mice. We also observe increased myeloid populations with a marker profile resembling MDSCs upon deletion of *miR-146a* and subsequent reductions in the level of this population in DKO mice. CCL5 is elevated in the absence of *miR-146a*, indicating that the *miR-146a* regulatory network influences CCL5 expression. Previous work has shown that *Ccl5* is regulated by *miR-146a* through the NF-κB signaling pathway in mice (40), and direct targeting of *Ccl5* by *miR-146a* has been shown in human cells (22). We find that loss of CCL5, in the context of *miR-146a* deficiency, restores tumor protection despite sustained weight gain and inflammation. We propose a model whereby *miR-146a* functions to limit the expansion of dysfunctional CD8^+^ T cells that express CCL5 and seem to be Taa cells. We have seen that these cells expand to greater levels in the absence of *miR-146a* and during aging, suggesting that age may predispose individuals to HCC. Furthermore, by limiting CCL5 production, the recruitment of suppressive immune cell populations to HCC tumors is reduced, ultimately resulting in decreased tumor growth due to improved immune control. Future work will investigate this model by testing whether these dysfunctional CD8^+^ T cells and aberrant myeloid cells play a causal role in the mouse HCC model and by further verifying the identity of these cell populations as Taa cells and MDSCs, respectively.

This study has focused immunologically on CD8 T cells and MDSCs. It is important to note, however, that there are other immune populations that change with mouse genotype in DEN-CDHFD–treated mice (Supplementary Fig. S8). We chose to focus on CD8 T cells and MDSCs, given the clear changes in their levels seen via flow cytometry across mouse genotype/disease status, as well as the clear rationale for their potential contribution to disease, such as Taa cells being known to secrete high levels of CCL5. Going forward, we hope that data like our scRNA-seq in Supplementary Fig. S8 can serve as a starting point for future studies into other immune populations that may play a role in *miR-146a*–mediated protection.

Another open question to be investigated is which cellular sources of *miR-146a* are important for HCC protection. Beyond our patient data showing that *miR-146a* is downregulated in cancerous tissue (suggesting that hepatocyte-derived *miR-146a* may be protective), Supplementary Fig. S1 also shows that in mice, normal hepatocytes, CD8^+^ T cells, and CD11b^+^ myeloid cells (a population that includes MDSCs) all express *miR-146a*. With expression across these cell types, any or all of them could contribute to the protective effect of *miR-146a*. To fully understand the mechanism of *miR-146a* protection, it will be essential for future work to investigate the role of various cellular sources in the protective phenotype.

Previous reports have shown anticorrelations between *miR-146a* expression and HCC (14, 28); our work builds on these findings by demonstrating a causal relationship between *miR-146a* and reduced tumor burden. To our knowledge, we are the first to address the role of *miR-146a* in liver cancer using a genetic mouse model of *miR-146a* deficiency. Furthermore, we show that *miR-146a*^*−/−*^*Ccl5*^*−/−*^ DKO mice regain protection against tumor formation. This provides mechanistic insight and suggests a role for *miR-146a* in regulating CCL5, a chemokine highly associated with liver cancer and disease (41, 42). This *miR-146a* genetic knockout, combined with a primary tumor model of HCC, provides a powerful understanding of the role of this anti-inflammatory miRNA in HCC. Furthermore, this genetic model provides a means to induce chronic inflammation in mice, which could be more broadly desirable for further study of liver disease, a condition that has previously been difficult to accurately model in a clinically relevant manner.

Another factor to consider about *miR-146a* protection is the potential for *miR-146a* targets beyond *Ccl5* to influence our phenotype. This is supported by the literature, in which other groups have identified *miR-146a as a* tumor suppressor in HCC through mechanisms involving *miR-146a* targets such as TRAF6 (14). Although it is essential to consider other potentially important targets, we still believe that the clear and dramatic reversal of phenotype observed with *Ccl5* KO suggests that this gene is critically important in *miR-146a*–mediated tumor protection. Additionally, it is plausible that other *miR-146a* targets, such as TRAF6 and IRAK1, contribute to increased tumor burden by influencing CCL5 expression. For example, NF-κB signaling is promoted by TRAF6 and IRAK1 and has been shown to trigger CCL5 expression (43).

Of note, although deletion of *Ccl5* restored the *miR-146a* HCC phenotype back to control levels, the gross metabolic phenotypes observed in *miR-146a*^*−/−*^ HCC mice remained despite the absence of CCL5. This indicates that *miR-146a* regulates metabolic and tumor growth phenotypes through two parallel and distinct pathways, implying that distinct targets of *miR-146a* protect against these pathologies. *miR-146a* repression of CCL5, which controls the *miR-146a* tumor growth phenotype, seems to occur through direct targeting of *Ccl5* or via IFNg-induced Stat1 and NF-κB signaling, which have been shown to be directly targeted by *miR-146a* (10, 44). Alternatively, the metabolic phenotype is likely regulated by macrophage functions via Traf6 targeting (9) or direct control of hepatocyte metabolic function through targets like MED1 (45). These results suggest that certain aspects of MASH can be uncoupled from HCC tumor phenotypes and that *miR-146a* is a multifaceted regulator of liver pathologies.

Sex disparity exists in HCC, with males having two- to fourfold higher rates than females (46). Perhaps because of this reduced prevalence, liver cancer in females has been understudied. This reduced prevalence is also observed in murine liver cancer models (27, 47, 48), and thus, females are often disregarded in HCC studies using animal models. Here, we show that *miR-146a* expression in females protects WT mice from HCC, with *miR-146a* deletion exacerbating disease penetrance in females. It has been demonstrated that differences in IL6 production between males and females contribute to some of the sex differences observed in DEN-induced HCC (27). This finding is consistent with our results, which show heightened IL6 production in *miR-146a*^*−/−*^ animals. Notably, IL6 also contributes to MDSC recruitment, which may also contribute to the miR-146a HCC phenotype. Given the known role of IL6 expression in HCC sex differences and our observation of IL6 differences in our model, this likely contributes to some of the sex differences that we observe. Another important note is that although less dramatic, tumor burden was increased in male *miR-146a*^*−/−*^ mice at 7 months of age (Supplementary Fig. S3), suggesting that miR-146a protection is at play in males, albeit to a lesser degree. The relevance of miR-146a-mediated protection in males is something of interest in further studies examining the efficacy of miR-146a as a therapeutic.

Because of the central role of both *miR-146a* and CCL5 in liver disease, these factors are candidates as targets for therapies to mitigate HCC. Notably, liver cells avidly uptake miRNAs and synthetic miRNA mimics, which have demonstrated strong clinical potential (49). Furthermore, CCL5 receptor inhibitors have also been utilized in preclinical settings. For instance, cenicriviroc, a CCR2/CCR5 inhibitor, significantly reduces monocyte and macrophage recruitment to the liver (50, 51). Moreover, the CCR5 antagonist maraviroc reduces macrophage infiltration into the liver, and treatment with this drug reduces overall tumor burden (52). Our work highlights the potential of CCL5 and *miR-146a* as therapeutic targets for HCC in the future, as well as *miR-146a* as a biomarker for this disease. As we continue to examine cell-specific roles for *miR-146a* and CCL5 in this context, we will be able to optimize therapeutic approaches to precisely target these molecules as a treatment for HCC.

## Supplementary Material

Figure S1 qPCR data showing miR-146a/miR-15b expression in mouse CD8s, myeloid cells, hepatocytes, and human liver

Figure S2 Mice treated with PBS vehicle control as opposed to DEN.

Figure S3 Tumor burden in male mice at 7 months.

Figure S4 Liver pathology analysis by histology in male mice and PBS-vehicle treated male and female mice.

Figure S5 Kuppfer and dendritic cell levels in liver of female DEN-CDHFD treated mice by flow.

Figure S6 Single cell anaylsis of CD8s showing elevated Taa marker expression.

Figure S7 CCL5 expression in CD8s and Taas of DEN-CDHFD treated mice.

Figure S8 Single cell data showing levels of various immune cells in DEN-CDHFD treated mice.

Supplementary Table 1 qPCR primers used in experiments

Supplementary Table 2 Flow cytometry antibodies used

Supplementary Table 3 Raw Data. Individual data points for all charts in main and supplemental figures, with the exception of scRNAseq data which is in the GEO database.

## Data Availability

The data generated in this study are available within the article and its supplementary data files. The scRNA-seq data were deposited into the NCBI Gene Expression Omnibus database (RRID:SCR_005012) at GSE138222 (Fig. 5; Supplementary Fig. S6) and GSE300674 (Fig. 7; Supplementary Fig. S8). Supplementary Table S3 has the raw data points for each figure.
