# Supplementary figures and images for: MicroRNA-146a Protects against Hepatocellular Carcinoma through Suppression of CCL5
Source: Cancer Res Commun. 2026 Feb 20;6(2):359–73. doi: 10.1158/2767-9764.CRC-25-0474 (PMC13138226; doi:10.1158/2767-9764.CRC-25-0474)

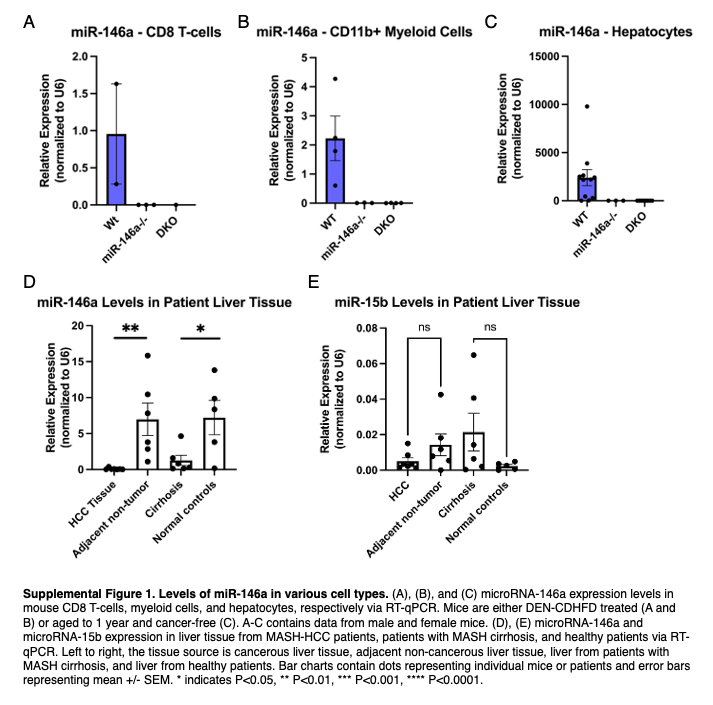

Supplement: Figure S1 — qPCR data showing miR-146a/miR-15b expression in mouse CD8s, myeloid cells, hepatocytes, and human liver [file crc-25-0474_figure_s1_suppsf1.png]

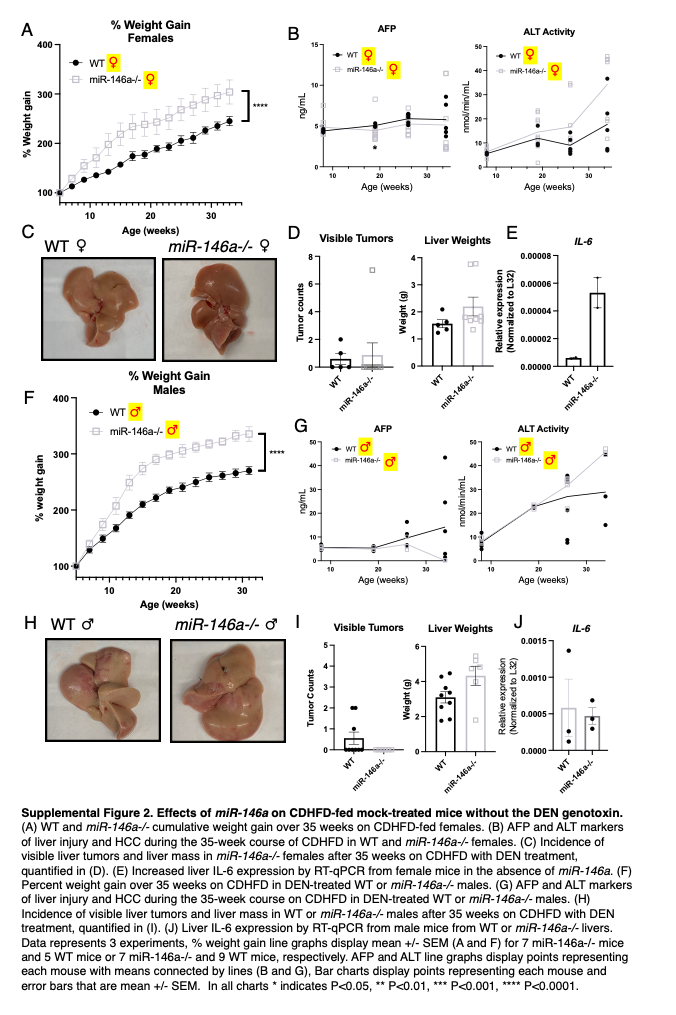

Supplement: Figure S2 — Mice treated with PBS vehicle control as opposed to DEN. [file crc-25-0474_figure_s2_suppsf2.png]

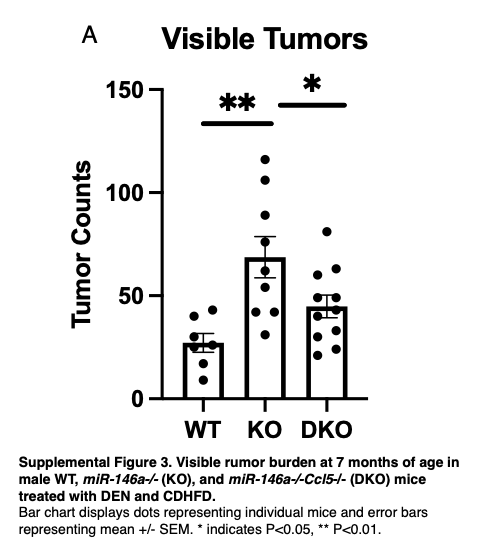

Supplement: Figure S3 — Tumor burden in male mice at 7 months. [file crc-25-0474_figure_s3_suppsf3.png]

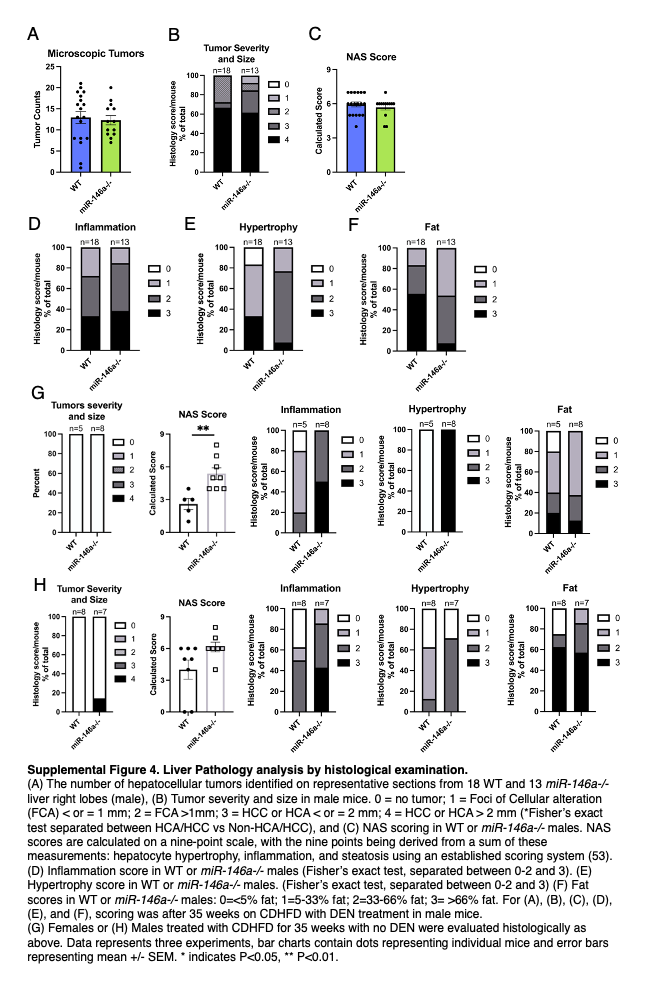

Supplement: Figure S4 — Liver pathology analysis by histology in male mice and PBS-vehicle treated male and female mice. [file crc-25-0474_figure_s4_suppsf4.png]

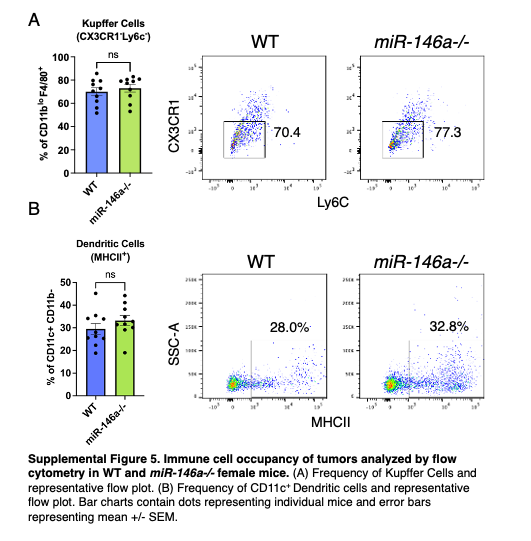

Supplement: Figure S5 — Kuppfer and dendritic cell levels in liver of female DEN-CDHFD treated mice by flow. [file crc-25-0474_figure_s5_suppsf5.png]

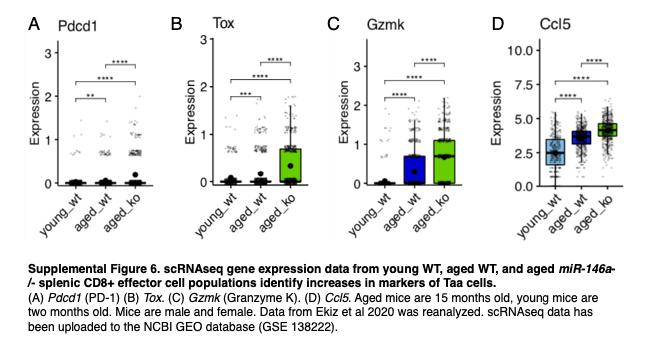

Supplement: Figure S6 — Single cell anaylsis of CD8s showing elevated Taa marker expression. [file crc-25-0474_figure_s6_suppsf6.png]

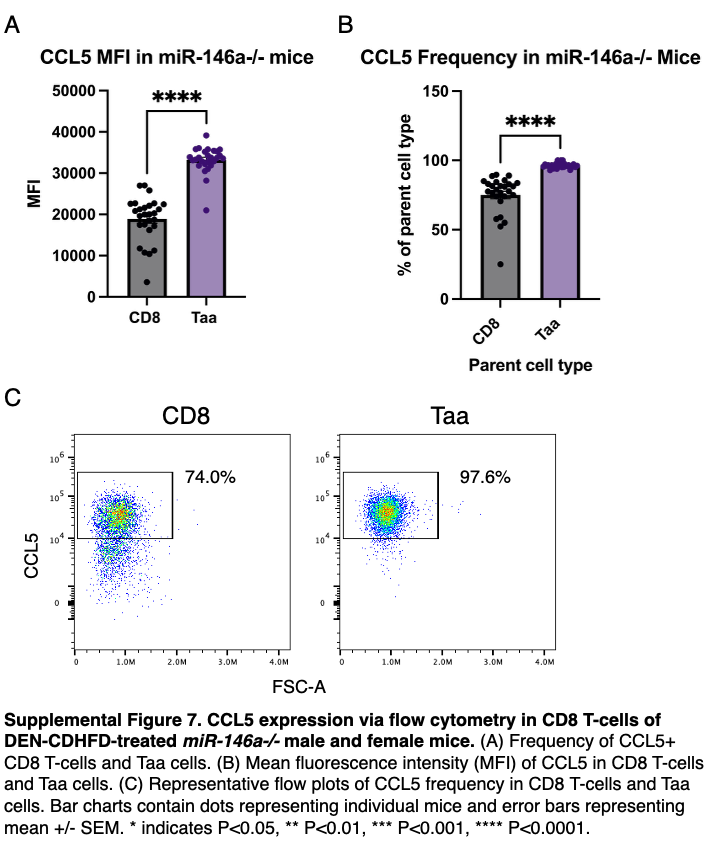

Supplement: Figure S7 — CCL5 expression in CD8s and Taas of DEN-CDHFD treated mice. [file crc-25-0474_figure_s7_suppsf7.png]

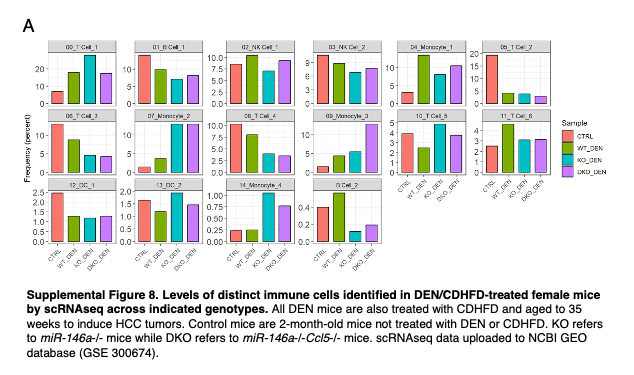

Supplement: Figure S8 — Single cell data showing levels of various immune cells in DEN-CDHFD treated mice. [file crc-25-0474_figure_s8_suppsf8.png]
